# Diagnosis and Management of Congenital H-Type Tracheoesophageal Fistula: Results of a National Survey

**DOI:** 10.3390/children11040423

**Published:** 2024-04-02

**Authors:** Cecilia Morchio, Alba Ganarin, Andrea Conforti, Ernesto Leva, Giovanni Gaglione, Gaia Brenco, Elisa Zambaiti, Salvatore Fabio Chiarenza, Tamara Caldaro, Maurizio Cheli, Giovanni Boroni, Elena Sofia Marcandella, Giovanna Riccipetitoni, Sebastiano Cacciaguerra, Vincenzo Di Benedetto, Valerio Gentilino, Gabriele Lisi, Francesco Morini, Paola Midrio

**Affiliations:** 1 School of Pediatric Surgery, University of Florence, 50100 Florence, Italy; cecilia.morchio@unifi.it; 2 Pediatric Surgery Unit, Ca’ Foncello Hospital, 31100 Treviso, Italy; alba.ganarin@aulss2.veneto.it; 3 Neonatal Surgery Unit, Medical and Surgical Department of Fetus-Newborn-Infant, Bambino Gesù Children’s Hospital, IRCCS, 00100 Rome, Italy; andrea.conforti@opbg.net; 4 Pediatric Surgery Unit, Fondazione IRCCS Ca’ Granda—Ospedale Maggiore Policlinico, University of Milan, 20100 Milan, Italy; ernesto.leva@policlinico.mi.it; 5 UOC Pediatric Surgery Unit, AORN Santobono-Pausilipon, 80100 Naples, Italy; giovannigaglione@tin.it; 6 Pediatric Surgery Unit, IRCCS Giannina Gaslini’s Hospital, 16100 Genova, Italy; gaia.brenco@gmail.it; 7 Department of Pediatric General Surgery, Regina Margherita Children’s Hospital, Azienda Ospedaliero Universitaria Città della Salute e della Scienza, 10100 Turin, Italy; ezambaiti@cittadellasalute.to.it; 8 Department of Pediatric Surgery, San Bortolo Hospital, 36100 Vicenza, Italy; fabio.chiarenza@aulss8.veneto.it; 9 Digestive Endoscopy and Surgery Unit, Bambino Gesu Children’s Hospital, IRCCS, 00100 Rome, Italy; tamara.caldaro@opbg.net; 10 Pediatric Surgery Unit, Ospedale Papa Giovanni XXIII, 24100 Bergamo, Italy; mcheli@asst-pg23.it; 11 Department of Paediatric Surgery, ASST Spedali Civili di Brescia, 25100 Brescia, Italy; giovanni.boroni@unibs.it; 12 Paediatric Surgery Unit, Women’s and Children’s Health Department, University of Padua, 35100 Padua, Italy; elenasofia.marcandella@aopd.veneto.it; 13 Department of Paediatric Surgery, “V. Buzzi” Children’s Hospital, 20100 Milan, Italy; giovanna.riccipetitoni@unipv.it; 14 Department of Clinical, Surgical, Diagnostic and Pediatric Sciences, University of Pavia, Fondazione IRCCS Policlinico San Matteo, 27100 Pavia, Italy; 15 Department of Pediatric Surgery, Ospedale Garibaldi-Nesima, 95100 Catania, Italy; sebcacci@gmail.com; 16 Department of Pediatric Surgery, G. Rodolico—San Marco Hospital, 95100 Catania, Italy; vdb@chirpedunict.it; 17 Division of Pediatric Surgery, Woman and Child Department, “Filippo Del Ponte” Hospital, ASST Sette Laghi, 21100 Varese, Italy; valerio.gentilino@asst-settelaghi.it; 18 Pediatric Surgery Unit, Santo Spirito Hospital, University of Chieti-Pescara, 65100 Pescara, Italy; gabriele.lisi@unich.it; 19 Department of Maternal and Child Health and Urological Sciences, La Sapienza University, 00100 Rome, Italy; francesco.morini@uniroma1.it

**Keywords:** congenital tracheoesophageal fistula, h-type esophageal atresia, type E esophageal atresia

## Abstract

Background: Congenital h-type tracheoesophageal fistula (H-TEF) without esophageal atresia (EA) represents about 4% of congenital esophageal anomalies. The diagnosis is challenging, and surgery is considered curative. The aim was to report a national survey on the diagnosis, management, and outcome of patients with congenital H-TEF. Methods: Following approval of the Italian Society of Pediatric Surgery, a survey was sent to all Pediatric Surgery Units to retrospectively collect H-TEF treated in the period 2010–2022. Descriptive analysis was performed, and results are given as prevalence, mean ± standard deviation (SD), or median and interquartile range (IQR). Results: The survey was sent to 65 units. Seventeen responded with one or more cases; 78 patients were diagnosed with H-TEF during the study period. Associated malformations were present in 43%, mostly cardiac (31%). The most frequent symptoms were cough (36%), bronchopneumonia (24%), and dysphagia (19%). H-TEF was detected by tracheobronchoscopy (90%), and/or upper GI (58%), and/or esophagoscopy (32%). The median age at diagnosis was 23 days (1 day–18 years). The most common approach was cervicotomy (76%), followed by thoracoscopy (14%) and thoracotomy (9%). The fistula underwent ligation and section of the fistula in 90% of the patients and clip closure and section in 9%. In one patient, the fistula was cauterized endoscopically. H-TEF preoperative cannulation was performed in 68% of cases, and a drain was placed in 26%. One month after surgery, 13% of the patients had mild persisting symptoms, mainly hypophonia. Recurrence occurred in 5%, and a second recurrence occurred in 1%. Conclusions: H-TEF prevalence was six cases/year, consistent with the expected rate of five cases/year in our country. The diagnosis was challenging, sometimes delayed, and, in most patients, required multiple examinations. Fistula ligation and section through cervicotomy were the most frequent treatment. Long-term outcomes are good, and recurrence is a rare event.

## 1. Introduction

In the pediatric population, tracheoesophageal fistula (TEF) is an uncommon condition and can either be congenital or acquired. The H-type tracheoesophageal fistula (H-TEF) without EA, also known as type E according to Gross classification [1], was first described by Lamb in 1873 as a variant of the congenital esophageal anomaly [2]. It occurs with an incidence of approximately 4% of all cases of EA. Unlike Gross type C, in which the fistula is normally found close to the carina, in type E, the fistula is more commonly located in the cervical region or thoracic inlet, usually making an oblique anteroposterior and cranio–caudal path. Patients with H-TEF may present other associated anomalies, although less frequently than other types of EA/TEF [3]. Diagnosis is invariably post-natal, and the clinical onset is often non-specific, consisting of choking during feeding, dysphagia, recurrent pneumonia, cyanosis, drooling, and weight loss, with the normal passage of a nasogastric tube. The classic triad of presenting symptoms first described by Helmsworth and Pryles (coughing during feeding, recurrent chest infection, and abdominal distension) is not always present [4]. Symptoms may be intermittent, leading to delayed diagnosis, even in late childhood or adulthood [5,6,7]. Once the diagnosis is suspected, tracheobronchoscopy (TBS), esophagoscopy, and/or upper gastrointestinal (GI) contrast studies are performed to confirm the presence and localization of the fistula. Since the first surgical repair in 1939 [8], surgery has become the mainstay of treatment. Even though different approaches were described, it seems that fistula closure using a cervical incision is the preferred one [9]. Due to its extreme rarity, only a few studies report large series in the literature [10,11,12,13]. The purpose of this study is to report a nationwide experience of diagnosis and treatment of congenital H-TEF.

## 2. Materials and Methods

We performed a national multi-institutional retrospective study of children with H-TEF. A survey, approved by the Italian Society of Pediatric Surgery (SICP), was distributed to all 65 Italian pediatric surgery centers. Each center was requested to provide data on patients aged 0–18 years old treated for congenital H-TEF between 2010 and 2022. The survey consisted of a standard data collection sheet divided into ten sections regarding reference hospital and demographics, prenatal diagnosis, birth data, associated malformations, symptoms at presentation, diagnostic work-up, surgery, recurrence, treatment in case of recurrence, and outcome. Each section was structured with a variable number of multiple-choice questions or open-ended questions. All answer sheets were collected, and data were organized by two dedicated surgeons (CM and AG). In case of incomplete, missing, or doubtful answers, the representative surgeon of the center was directly contacted. Data regarding patients not diagnosed during the period 2010–2022 or older than 18 years old or with a non-congenital isolated TEF were excluded. We conducted a descriptive statistical analysis, with the results presented as prevalence, mean ± standard deviation (SD), or median and interquartile range (IQR) for normally or not normally distributed data, respectively.

The ethics committee approval was deemed unnecessary primarily because the database we used was anonymous and each center retained its patients’ names, and secondly, because of the retrospective nature of the work.

## 3. Results

Nineteen out of sixty-five centers responded, and seventeen of them reported at least one patient diagnosed with H-TEF during the study period. A total of 78 patients (40 males and 38 females) were collected with a median number of cases/centers of four (range 1–14). The mean gestational age at birth was 38 weeks ± 2 weeks. The mean birth weight was 2990 g ± 623. As expected, no case was diagnosed nor suspected during gestation. Thirty-three out of seventy-eight patients (42%) had at least one associated malformation, and twelve of them presented more than one (Table 1). Cardiac malformations were the most common (24/33, 72%), while the less frequent were anorectal and intestinal (6/33, 15% both). Four patients (5%) had Down syndrome, and 75% of them had another malformation in addition to TEF. The median age at diagnosis was 23 days (IQR = 10–74), with a very wide range from 1 day to 18 years. Of note, four patients (0.5%) were diagnosed during adolescence. The most frequent symptoms at presentation were cough (36%), bronchopneumonia (24%), and dysphagia (19%). Other reported symptoms included drooling (9%), desaturation (5%), and weight loss (1%). Regarding the diagnostic work-up, neither instrumental nor radiological technique was 100% successful in detecting the fistula. Tracheobronchoscopy (TBS), performed in 70 patients (90%), identified the fistula in 66/70 cases (94.3%). Esophagogastroscopy, performed in 25 patients (32%), detected the fistula in 21/25 (84%) cases. Finally, the upper GI contrast study, performed in 45 patients (58%), revealed the fistula in 30/45 (66.6%) cases. Median age at surgery was 31 days (IQR = 16–82). Pre-operatively, a probe was inserted in the fistula in 68% of the cases. Closure of the fistula was performed through cervicotomy (76%), thoracoscopy (14%), thoracotomy (9%), or endoscopy (1%). At the first operation, the fistula was ligated and sectioned in 70 patients, closed with clips, and sectioned in 7 or endoscopically sclerotized in 1. A drain was placed in 26% of the procedures. No intraoperative complication was reported. One patient with multiple malformations, born at 33 weeks of gestational age with a birth weight of 900 g, was diagnosed and treated at 7 days of life but died the day after surgery. One month after surgery, 13 patients (16%) had mild persisting symptoms, such as hypophonia (10 patients), dysphagia (2), and bronchopneumonia (1). One year after surgery, mild hypophonia was still present in three patients, occasional cough episodes in three, and dysphagia in one. Otherwise, all patients are thriving and in good general condition. Recurrence occurred in 4/78 patients (5%) (Table 2), with one patient experiencing two recurrences. In these patients, the initial surgical approach was thoracoscopic clip closure and section (2/4), ligation and section through cervicotomy (1/4), and endoscopic sclerosis (1/4). The first recurrence was treated by ligation and section through cervicotomy (2), thoracotomy (1), or endoscopic sclerosis (1). The second recurrence occurred after the endoscopic approach and was eventually treated by ligation and section through cervicotomy.

## 4. Discussion

Congenital H-TEF is an extremely rare condition that represents about 4% of the EA/TEF spectrum. In our series, the incidence of H-TEF was six cases/year, consistent with the expected rate of five cases/year in our country. Only a few centers responded (19/65), and only 17 reported H-TEF patients, which may reflect the referral of this rare disease from small to high-volume hospitals. Even though congenital H-TEF seems to have a lower association with other anomalies [3], in our population, these were present in 42% of cases, with cardiac malformations being the most prevalent (31%). In two similar series, cardiac malformations were described in approximately 20% of cases [10,13]. Four patients in our series (5.1%) had Down syndrome, in line with previous reports [14]. Patients born with congenital H-TEF often present with respiratory distress and feeding difficulty shortly after birth. These symptoms result from the passage of air, saliva, and liquid food during feeding through the fistula. Accordingly, in our series, the most common symptoms were cough (36%), bronchopneumonia (24%), and dysphagia (19%). The classical triad [3] (coughing while feeding, abdominal distension, and pneumonia) was not reported in any of our cases. In a multicenter series of 23 patients, chest infection (70%), cough with feedings (52%), and cyanosis (43%) were found to be common presenting symptoms [15]. A multicenter review of 102 patients from 14 centers in the USA reported that choking while feeding was present in a large majority at presentation, either associated or not with desaturation [10]. In a retrospective series of 56 cases, cyanotic episodes (70%), choking with feeds (52%), and aspiration pneumonia (46%) were noted as the most common presenting symptoms [13]. Due to the non-specific symptoms, the diagnosis could be delayed. Only 55% of our patients (43/78) were symptomatic during the first week of life. This figure differs from that reported in other studies: in two series, 84% [13] and 70% [16] of patients presented with symptoms during the first week of life. In our series, four patients (5%) were diagnosed during late adolescence, following a symptomatic history of recurrent bronchopneumonia episodes and chronic cough, in agreement with the literature [5,17,18,19]. Due to the potentially life-threatening implications associated with congenital H-TEF, precise and prompt diagnosis and subsequent treatment are imperative. Nevertheless, owing to the elusive nature of H-TEF, the diagnostic process often necessitates multiple examinations before a definitive conclusion can be drawn. This challenging diagnostic journey underscores the importance of vigilant clinical evaluation and the utilization of advanced diagnostic modalities to swiftly identify and address this condition.

Tracheobronchoscopy (TBS) and esophagoscopy are integral diagnostic procedures utilized to directly visualize and assess the presence, dimensions, and precise anatomical location of the fistulous tract. These endoscopic examinations play a pivotal role in accurately delineating the extent and characteristics of the fistula, facilitating informed decision-making regarding subsequent management strategies [20].

In terms of diagnostic assessment, it is imperative to rule out the presence of a fistula. The fistula’s location could be detected by conducting positive pressure distension concurrently with tracheobronchoscopy using a rigid ventilating bronchoscope. An interesting study conducted by Yasuda et al. in 2020 addressed the issue of missing tracheoesophageal fistulas using tracheobronchoscopy alone and proposed the potential utility of conducting a CO_2_ insufflation test during endoscopy to better detect the fistula [21].

In the present series, almost all patients (90%) underwent TBS, and in only 6%, the examination failed to detect the fistula, similar to what is reported in the literature [22]. In the upper GI contrast study, a contrast agent is swallowed or instilled through a nasoesophageal tube into the esophageal to visualize the fistula while the patient lies in the prone position. Comparing our experience with the literature, it appears that the H-TEF could often be missed with upper GI contrast studies. TEF was undetected after esophageal contrast examinations in up to 50% of cases in other series [12,15] and in 33% of present cases. Regarding the operative treatment, in the present collected series, cervicotomy is the most used approach (76% in our series), which is similar to the available literature. In a 2014 review, considering 17 different studies describing patients who underwent open surgical procedures, the cervical approach was performed in 90% of cases [9]. In 2017, a systematic multicenter review involving a cohort of 102 patients across 14 centers reported that 96% of cases were successfully addressed using the cervical approach, leaving a minority managed through the thoracic approach [10]. Furthermore, in an extensive single-center series spanning from 1948 to 2017, including 56 patients, the cervical approach was used in 90% of the cases [13].

**Figure 1:**
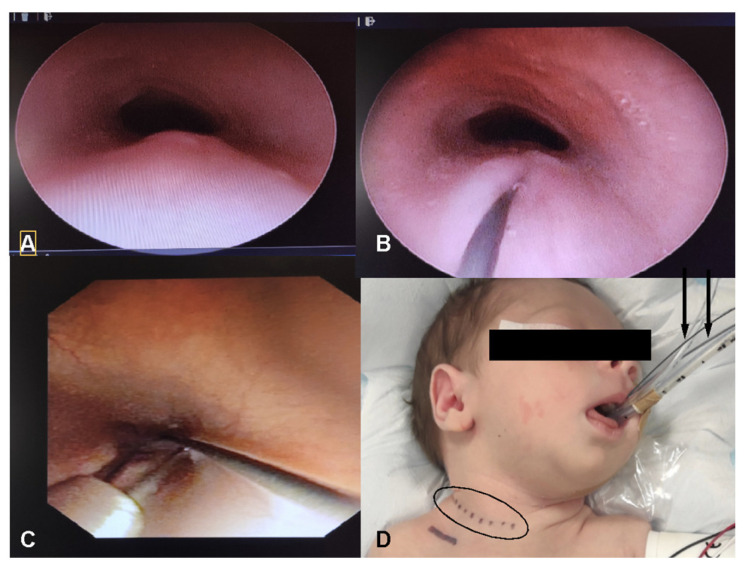
Tracheoesophageal fistula is recognized during tracheoscopy (bulging in the posterior tracheal wall) (**A**). The fistula is cannulated (**B**), and then an esophagoscopy is performed to retrieve the wire (**C**). Right cervicotomy (black dashed line) is performed (**D**); the guide wire (black arrows), exiting from the mouth, helps recognize the tracheoesophageal fistula.

In the decision-making process, significant consideration should be given to the utilization of a radio-opaque wire passage through the fistulous tract. This technique serves a crucial role in precisely delineating the anatomical position of the fistula, thereby providing invaluable guidance for determining the most optimal surgical approach. By facilitating enhanced visualization and localization of the fistula, the passage of a radio-opaque wire not only aids in preoperative planning but also contributes to the successful execution of surgical interventions aimed at addressing congenital H-TEF (Figure 1).

In terms of post-operative complications, the most common issues documented in the literature involve airway problems, such as hypophonia, stridor, and intermittent cough. The source of airway-related symptoms can be attributed to injury of the recurrent laryngeal nerve, which is transient in most cases and reported to occur in 15% to 50% of patients [11,23]. Continuous recurrent laryngeal nerve monitoring has been proposed by some authors and may help identify the nerve during dissection [24]. As it is not uncommon for this problem to go unnoticed, some authors recommend performing post-operative routine vocal cord evaluation through laryngoscopy, aiming to identify unilateral or bilateral cord paralysis and establish a follow-up plan [11,25], although no established follow-up protocol exists. In our series, clinical follow-up at one month and one year after surgery revealed hypophonia, respectively, in 13% and 4% of patients.

Fallon et al. report that only 33% of their patients underwent laryngoscopy aimed to identify cord dysfunction, suggesting potential underestimation in their data [10].

Zani emphasizes the importance of a multidisciplinary approach along with the execution of a routine laryngoscopy in all post-operative cases because, although the majority of patients with cord paralysis are asymptomatic, two-thirds of them do not fully regain normal function [11]. In our case studies, instrumental follow-up was only performed on symptomatic patients. For the evaluation of vocal fold movement, we underscore the importance of conducting a flexible laryngoscopy in all patients before and after the surgical procedure. Pre-procedure assessment is essential as certain children may have congenital vocal fold movement impairment, which could impact the procedure or treatment plan. Secondly, post-procedure evaluation is equally important due to the high prevalence of this issue. The implementation of a standardized follow-up protocol is paramount, encompassing a post-operative endoscopic assessment via laryngoscopy, with provisions for repeat examinations in case of abnormalities.

Additionally, scheduled clinical evaluations at six- and twelve-month intervals are integral for monitoring overall recovery progress and identifying potential manifestations of dysphagia. Such a systematic approach not only aids in the early detection and management of asymptomatic complications but also enables meaningful comparisons across various patient cohorts, thereby enhancing the comprehensiveness and effectiveness of post-operative care protocols. Moreover, H-TEF patients should be centralized in third-level hospitals, given the rarity of the disease.

Within our cohort, a notable recurrence was observed, with four patients (constituting 5% of the total) experiencing a recurrence of TEF, including one individual with dual recurrence episodes. Notably, within the dataset under review, endoscopic interventions for the management of H-TEF, whether employed initially or after a recurrence, proved ineffective. Although the incidence of such occurrences remains exceedingly low, these findings warrant contemplation and may prompt further investigation into the efficacy of current therapeutic modalities for managing recurrent H-TEF cases.

A systematic review from 2021 identified a recurrence rate ranging between 2% and 3%, regardless of the type of intervention performed during the initial treatment [23]. Cuestas et al. reported a case series where a patient underwent three recurrences, all treated with sclerosis, eventually solved by means of surgery [26]. Endoscopic closure may appear attractive because of the minimal invasiveness. On this issue, the literature is contradictory: Daniel et al. indicate an elevated incidence of fistula recurrence after this approach [27], while in a recent systematic review and meta-analysis, the endoscopic technique is suggested as a first-line approach, even if approximately two procedures must be anticipated to achieve the complete treatment [28]. In a recent multicenter study, the authors suggest performing an open approach in cases of H-type fistula recurrences, given the limited utilization of the endoscopic approach, which yielded suboptimal outcomes in a few cases [29].

In order to prevent the recurrence of tracheoesophageal fistulas, some authors recommend a tracheopexy to avoid this complication [24].

The study’s strength lies in its comprehensive national scope, which, despite the low response rate, likely provides a representation of the actual incidence of H-TEF. Additionally, the moderately extended follow-up duration contributes to suggesting the typically benign nature of this rare condition. However, there are certain limitations to consider. These include the small sample size, which is inherent to the rarity of the condition studied. The low response rate to the survey, possibly influenced by the absence of smaller centers participating, might imply a lack of patients seen or their transfer. Finally, the study’s retrospective design should also be acknowledged as a potential limitation.

## 5. Conclusions

H-TEF, being an exceptionally rare congenital anomaly, demands a vigilant approach from pediatric surgeons due to the non-specific nature of their symptoms, which often mimic those of other conditions. This necessitates a high level of suspicion and careful consideration during diagnosis and treatment.

Diagnosing H-TEF is notably challenging and often involves multiple examinations, leading to potential delays in identification. Among these diagnostic methods, tracheobronchoscopy (TBS) seems to be the most reliable one. Surgical closure and section, through the appropriate anatomical approach, is curative, and recurrence is a rare event. Although airway issues such as hypophonia and stridor are the most frequent post-operative complications, outcomes are usually favorable.

## Figures and Tables

**Table 1:** Associated malformations.

Type of Malformation	N. Patients (%)
Cardiac	24 (72%)
Genitourinary	9 (27%)
Vertebral	7 (21%)
Intestinal	6 (18%)
Ano-rectal	6 (18%)

**Table 2:** Surgical treatment in 4 patients with TEF recurrence.

First Surgery	Second Surgery	Third Surgery
sclerosis (endoscopy)	ligation + section (cervicotomy)	
clips + section (thoracotomy)	ligation + section (thoracotomy)	
clips + section (thoracoscopy)	sclerosis (endoscopy)	ligation + section (cervicotomy)
ligation + section (cervicotomy)	ligation + section (cervicotomy)	

## Data Availability

The data presented in this study are available on request from the corresponding author.
